# Upregulated Connexin 43 Induced by Loss-of-Functional S284L-Mutant α4 Subunit of Nicotinic ACh Receptor Contributes to Pathomechanisms of Autosomal Dominant Sleep-Related Hypermotor Epilepsy

**DOI:** 10.3390/ph13040058

**Published:** 2020-03-29

**Authors:** Kouji Fukuyama, Masashi Fukuzawa, Ruri Okubo, Motohiro Okada

**Affiliations:** 1 Department of Neuropsychiatry, Division of Neuroscience, Graduate School of Medicine, Mie University, Tsu, Mie 514-8507, Japan; k-fukuyama@clin.medic.mie-u.ac.jp (K.F.); ddduck0602@gmail.com (R.O.); 2 Department of Biology, Faculty of Agriculture and Life Science, Hirosaki University, Hirosaki 036-8560, Japan; fukuzawa@hirosaki-u.ac.jp

**Keywords:** idiopathic epilepsy, zonisamide, microdialysis, connexin, hemichannel

## Abstract

To study the pathomechanism and pathophysiology of autosomal dominant sleep-related hypermotor epilepsy (ADSHE), this study determined functional abnormalities of glutamatergic transmission in the thalamocortical motor pathway, from the reticular thalamic nucleus (RTN), motor thalamic nuclei (MoTN) tosecondary motor cortex (M2C) associated with the S286L-mutant α4β2-nicotinic acetylcholine receptor (nAChR) and the connexin43 (Cx43) hemichannel of transgenic rats bearing the rat S286L-mutant *Chrna4* gene (S286L-TG), which corresponds to the human S284L-mutant *CHRNA4* gene using multiprobe microdialysis, primary cultured astrocytes and a Simple Western system. Expression of Cx43 in the M2C plasma membrane fraction of S286L-TG was upregulated compared with wild-type rats. Subchronic nicotine administration decreased Cx43 expression of wild-type, but did not affect that of S286L-TG; however, zonisamide (ZNS) decreased Cx43 in both wild-type and S286L-TG. Primary cultured astrocytes of wild-type were not affected by subchronic administration of nicotine but was decreased by ZNS. Upregulated Cx43 enhanced glutamatergic transmission during both resting and hyperexcitable stages in S286L-TG. Furthermore, activation of glutamatergic transmission associated with upregulated Cx43 reinforced the prolonged Cx43 hemichannel activation. Subchronic administration of therapeutic-relevant doses of ZNS compensated the upregulation of Cx43 and prolonged reinforced activation of Cx43 hemichannel induced by physiological hyperexcitability during the non-rapid eye movement phase of sleep. The present results support the primary pathomechanisms and secondary pathophysiology of ADSHE seizures of patients with S284L-mutation.

## 1. Introduction

Until recently, numerous gene mutations of ion channels, which regulate neuronal excitabilities in the central nervous system, have been identified in the various pedigrees of idiopathic epilepsy syndromes. In vitro expression studies using Xenopus oocytes or human embryonic kidney cells have demonstrated the functional abnormalities of mutant ion channels, whereas these abnormalities cannot fully resemble the situation in vivo [1]. In contrast, animal models bearing the mutant gene corresponding to the human mutation have the advantage that the functional consequences of a given mutation can be studied in the complex context of an intact living organism [1].

Autosomal dominant sleep-related hypermotor epilepsy (ADSHE) [2] was first identified as distinct familial idiopathic epilepsy (previously ADNFLE: autosomal dominant nocturnal frontal lobe epilepsy) in 1994 [3]. Typically, ADSHE seizures are symptomatically comparable to those seen in frontal lobe epilepsy and occur predominantly during the non-rapid eye movement sleep phase [2,4,5,6]. These seizures are complex, stereotyped hyperkinetic seizures that consist of three types of motor seizures, ‘nocturnal paroxysmal arousals’, ‘nocturnal paroxysmal dystonia’ and ‘episodic nocturnal wandering’ [2,4,5,6]. Onset ages of ADSHE are usually prior to age 20 (mean age of onset is 14 years) [2,4,5,6]. Although the majority of ADSHE patients are not progressive but lifelong, frequencies of ADSHE seizures are usually reduced age-dependently. Interestingly, a sporadic form of sleep-related hypermotor epilepsy (SHE) [2,7] (previously NFLE: nocturnal frontal lobe epilepsy), is clinically indistinguishable from those of ADSHE [4,5]. Therefore, any observed clinical phenotypes have been considered to belong uniformly to ADSHE/SHE syndrome [2].

The seizure features of ADSHE/SHE syndrome are mostly uniform; however, ADSHE/SHE syndrome is classified based on the characteristics in two major clinical features, antiepileptic drug sensitivity and comorbidity with cognitive deficit. In spite of the uniformity of ADSHE/SHE syndrome, various missense mutations in genes encoding α2 subunit (*CHRNA2*) of the nicotinic acetylcholine receptor (nAChR), α4-nAChR (*CHRNA4*), β2-nAChR (*CHRNB2*), corticotropin-releasing hormone (*CHR*), sodium-activated potassium channel subunit 1 (*KCNT1*) and DEP domain containing protein 5 (*DEPDC5*) have been identified in many ADSHE pedigrees [2,5,6]. Several clinical studies reported that gene mutations provide the variances in ADSHE/SHE syndrome, since the classical three ADSHE mutations of *CHRNA4*, S280F, S284L and insL, differ significantly with respect to their neuropsychiatric comorbidities and antiepileptic drug sensitivities. It has been well established that carbamazepine (CBZ) is the first-choice anticonvulsant for ADSHE/SHE and leads to remission in approximately 60% of ADSHE/SHE patients, including ADSHE patients with S280F and insL mutations [4,8,9]. However, ADSHE patients with S284L mutation are resistant to CBZ but responsive to other anticonvulsants such as zonisamide (ZNS) [5,10,11,12,13]. Usually ADSHE/SHE seizures are the sole major symptom of ADSHE/SHE syndrome, and additional neuropsychiatric features have been reported in just lower than 3% of ADSHE patients with S280F mutation [6,14,15,16,17,18]. Contrary, ADSHE patients with insL and S284L mutation have been known to be comorbid with cognitive disturbances such as schizophrenia-like psychosis, autism and intellectual disability [7,11,12,13,16,19,20]. Therefore, ADSHE is a syndrome of heterogeneous aetiologies characterized by the occurrence of sleep-related seizures with various motor manifestations, depending on the involved functional abnormalities of neuronal networks within the frontal cortex and its associated neural circuits. Indeed, there are intra- and inter-familial variations in both onset age and prognosis in the same ADSHE pedigree [2,4,5,6].

Recently, we demonstrated the pathophysiology of CBZ-resistant/ZNS-sensitive ADSHE seizures and pathomechanisms of comorbidity of cognitive deficits using a genetic ADSHE model rat, namely the S286L transgenic rat (S286L-TG), bearing the missense S286L mutation in the rat *Chrna4* gene which corresponds to the S284L mutation in the human *CHRNA4* gene [21,22]. These studies using S286L-TG demonstrated several pathomechanisms of ADSHE seizures associated with the S284L mutation [21,22]. The first was that the basal extracellular L-glutamate level in various brain regions of S286L-TG were larger compared with wild-type rats [21,22], similar to S284L-TG [23]. The second was that activation of S286L-mutant α4β2-nAChR in the reticular thalamic nucleus (RTN) of S286L-TG generated the relatively GABAergic disinhibition in the motor thalamic nuclei (MoTN) resulting in enhancement of glutamatergic transmission in the thalamocortical motor pathway, from the MoTN to the secondary motor cortex (M2C) [21], and in the thalamic hyperdirect pathway, from the MoTN to the subthalamic nucleus (STN) [22]. The third was that the M2C itself cannot independently generate epileptic discharge, whereas the M2C can integrate external excitatory inputs from the thalamocortical motor pathway (MoTN-M2C pathway), resulting in the generation of epileptic discharges in the M2C [21]. These findings suggest the pathomechanisms of three major ADSHE seizures, ‘nocturnal paroxysmal arousals’, ‘nocturnal paroxysmal dystonia’ and ‘episodic nocturnal wandering’ [21,22].

In spite of these efforts, additional studies are needed to clarify the pathomechanisms and pathophysiology of ADSHE with the S284L mutation. Exploring the detailed mechanisms of enhanced basal extracellular L-glutamate levels in the MoTN, M2C and STN of S286L-TG [21,22,23], as well as the mechanisms of focus generation due to the integration of hyperglutamatergic transmission of the thalamocortical motor pathway in the M2C [21,22], will contribute to breakthroughs in the elucidation of epileptogenesis and/or ictogenesis of ADSHE with the S284L mutation. Furthermore, even if ADSHE seizures are controlled, once a patient experiences an ADSHE seizure, they will experience many ADSHE seizures during that same night [4]. These intra-individual variations of ADSHE seizure frequency suggest the possibilities that the event-related factors leading to epileptogenesis and/or ictogenesis of ADSHE probably play important roles in the secondary pathogenesis of ADSHE seizures.

In vitro experiments revealed that α4β2-nAChR activation can decrease endothelial intercellular communication via the downregulation of connexin 43 (Cx43) [24]. Connexin is a family of 21 protein isoforms, and 11 connexin isoforms are expressed in the central nervous system [25,26,27]. Six connexin proteins assemble to form a homomeric/heteromeric connexon. Two connexon in two neighboring cells (including neuron, astrocyte, oligodendrocyte and microglia) form a gap-junction channel with an aqueous pore and charged surface walls [25,26,27]. A single connexon contributes to chemical connection between intra- and extra-cellular spaces as hemichannels [25,26,27]. Cx43 is the most widely expressed and predominantly expressed isoform in the central nervous system, including in astrocytes [28,29]. Accumulating evidence indicates that connexin is crucial to the coordination and maintenance of physiologic activity including neuronal excitability, synaptic plasticity, tripartite synaptic transmission and homeostasis maintenance in the central nervous system [26,27]. Furthermore, the expression of Cx43 is increased in glia but not in neurone in animal models and patients with epilepsy [30]. In particular, inhibitors of connexin channels can prevent the onset of epileptic seizures [26,31,32]. Recently, we demonstrated that an atypical antipsychotic, clozapine, was able to increase hemichannel activity as well as Cx43 expression in a concentration dependent manner in the plasma membrane of astrocytes [33,34]. The stimulatory effects of clozapine on Cx43 expression however plays a double-edged sword where it has demonstrated improvement in cognitive dysfunction in antipsychotic-refractory schizophrenia but has also resulted in clozapine-induced toxic myocarditis and convulsion via enhanced astroglial L-glutamate release [33,34].

Based on these clinical and preclinical findings, to explore the pathomechanisms and pathophysiology of ADSHE seizure associated with Cx43 induced by S284L-mutant α4β2-nAChR, the present study determined the effects of the subchronic administration of a therapeutic-relevant dose of ZNS on functional abnormality of transmission in the focus region (M2C) and the thalamocortical motor pathway associated with Cx43 and α4β2-nAChR of S286L-TG. This study used multiprobe microdialysis and the effects of nicotine and zonisamide on Cx43 expression in plasma membrane of subchronically administrated S286L-TG and primary cultured astrocytes using a Simple Western system.

## 2. Materials and Methods 

### 2.1. Chemical Agents

The selective α4β2-nAChR agonist, (E)-N-Methyl-4-(3-pyridinyl)-3-buten-1-amine oxalate (RJR2403) was obtained from Cosmo Bio (Tokyo, Japan) [35]. Amino-3-(3-hydroxy-5-methyl-isoxazol-4-yl)propanoic acid (AMPA) and nicotine ditartrate [35] were obtained from Wako Chemicals (Osaka, Japan). The selective Cx43 inhibitor, GAP19 [33,36] was obtained from Funakoshi (Tokyo, Japan). Zonisamide sodium salt (ZNS) was provided by Dainippon-Sumitomo Pharma (Osaka, Japan). All compounds were prepared on the day of the experiment. RJR2403, AMPA, GAP19 and ZNS were dissolved in modified Ringer’s solution (MRS) or 100 mM potassium containing MRS (HKMRS) directly for microdialysis study. ZNS and nicotine were also dissolved in artificial cerebrospinal fluid (ACSF) or Dulbecco’s modified Eagle’s medium containing 10% fetal calf serum (fDMEM) for the primary cultured astrocyte study. The detailed compositions of ACSF, fDMEM, MRS and HKMRS are described in the following section.

Previous studies have reported that the chronic administration of a therapeutic-relevant dose of ZNS ranged from 25 mg/kg/day to 50 mg/kg/day [37,38,39]. The therapeutic-relevant plasma concentration of ZNS against epilepsy ranged from 47 to 330 μM [38,40]. According to previous reports, in the present study, to study the subchronic effects of ZNS, each rat was subcutaneously administered ZNS (40 mg/kg/day for 7 days) [23] using a subcutaneous osmotic pump (2ML_1, Alzet, Cupertino, CA; the nominal pumping rate and duration was 10 μL/h for 7 days). Primary cultured astrocytes were subchronically administrated the lowest therapeutic-relevant concentration of ZNS (50 μM).

To study the effects of nicotine on Cx43 expression in the plasma membrane, rats were subchronically administrated nicotine (10, 25 and 50 mg/kg/day) for 7 days using a subcutaneous osmotic pump (2ML_1, Alzet), according to a previous clinical study [41]. Primary cultured astrocytes were administered 1 and 3 μM nicotine for 7 days.

### 2.2. Preparation of the Microdialysis System

Animal care, the experimental procedures, and protocols for animal experiments were approved by the Animal Research Ethics Committee of the Mie University School of Medicine (No. 24–37-R1). All studies involving animals have been reported in accordance with the ARRIVE guidelines for reporting experiments involving animals [42]. A total of 204 rats were used in the experiments described. Male S286L-TG [21,22] and wild-type littermates were anesthetized with 1.8% isoflurane and then placed in a stereotactic frame. A concentric direct-insertion type dialysis probe (0.22 mm diameter, 2 mm exposed membrane: Eicom, Kyoto, Japan) was implanted in the secondary motor cortex (M2C: A = +1.8 mm, L = +1.6 mm, V = −2.4 mm, relative to bregma), motor thalamic nuclei comprising ventroanterior and ventrolateral thalamic nuclei (MoTN: A = −2.0 mm, L = +1.4 mm, V = −7.2 mm, relative to bregma), and reticular thalamic nucleus (RTN: A = −1.4 mm, L = +1.2 mm, V = −7.2 mm, relative to bregma) [43].

Perfusion experiments were commenced 18 h after recovery from isoflurane anaesthesia. The perfusion rate was set at 2 μL/min in all experiments, using modified Ringer’s solution (MRS) composed of the following (in mM): 145 Na^+^, 2.7 K^+^, 1.2 Ca^2+^, 1.0 Mg^2+^, and 154.4 Cl^−^, buffered with 2 mM phosphate buffer and 1.1 mM Tris buffer at pH 7.4, or 100 mM potassium containing MRS (HKMRS) composed of the following (in mM): 47.7 Na^+^, 100.0 K^+^, 1.2 Ca^2+^, 1.0 Mg^2+^, and 154.4 Cl^−^, buffered with 2 mM phosphate buffer and 1.1 mM Tris buffer at pH 7.4. The detailed experimental designs are described in the following section (in Section 2.3). Extracellular L-glutamate level was measured by ultra-high-performance liquid chromatography (UHPLC), 8 h after starting the MRS perfusion. When the coefficients of variation for L-glutamate reached < 5% over a period of 60 min (stabilization), control data were obtained over another 60 min period (pre-treatment period). This was followed by perfusion with MRS containing target agents. Following the microdialysis experiments, the rats were anesthetized using 1.8% isoflurane and their brains removed. The locations of the dialysis probes were verified by histological examination using 200 μm thick tissue slices (Vibratome 1000, Technical Products International, St. Louis, MO, USA).

### 2.3. Experimental Designs of Microdialysis Study

**Figure 1:**
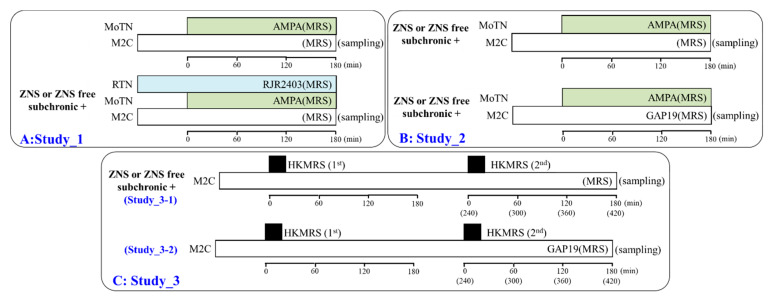
Schematic experimental designs of microdialysis experiments. Panels **A**, **B**, **C** are represented the experimental designs of Study_1, Study_2 and Study_3, respectively. MRS: modified Ringer’s solution, HKMRS: 100 mM potassium containing MRS, RJR2403 (100 μM: selective α4β2-nAChR agonist): (E)-N-Methyl-4-(3-pyridinyl)-3-buten-1-amine oxalate, AMPA (100 μM: AMPA/glutamate receptor agonist): amino-3-(3-hydroxy-5-methyl-isoxazol-4-yl)propanoic acid, GAP19 (100 μM: selective connexin43 (Cx43) hemichannel blocker), MoTN: motor thalamic nuclei, RTN: reticular thalamic nucleus, M2C: secondary motor cortex.

Rats were randomly assigned to the treatment groups of each experiment. The three experimental designs were distinguished by the route of drug administration. Experiments were not started until three consecutive baseline transmitter measurements yielded a coefficient of variation of less than 5%. Dialysates were then collected for 60 min (pretreatment period) followed by 180 min of sampling after the administration of experimental agents. The schematic experimental designs are shown in Figure 1.

#### 2.3.1. Study_1: Effects of Subchronic Administration of the Therapeutic-Relevant Dose of ZNS on Glutamatergic Transmission in the Thalamocortical Motor Pathway

Rats were subchronically administrated with or without therapeutic-relevant dose of ZNS (40 mg/kg/day) for 7 days [39,40,44] using a subcutaneous osmotic pump (2ML_1, Alzet, Cupertino, CA, USA). To explore the effects of subchronic administration of a therapeutic-relevant dose of ZNS on glutamatergic transmission in the thalamocortical motor pathway (RTN-MoTN-M2C) of wild-type and S286L-TG, the perfusion medium in the RTN was replaced with MRS with or without 100 μM RJR2403 (α4β2-nAChR agonist) (Figure 1A). The perfusates in the MoTN and M2C were commenced with MRS alone (Figure 1A). After stabilization of L-glutamate levels in the M2C, the perfusate in the MoTN was switched from MRS to MRS containing 100 μM AMPA (AMPA/glutamate receptor agonist) for 180 min (Figure 1A). During experiments, the perfusate in the M2C were maintained by MRS alone (Figure 1A).

#### 2.3.2. Study_2: Interaction between Subchronic Administration of the Therapeutic-Relevant Dose of ZNS and Acute Local Administration of GAP19 into the M2C on Glutamatergic Transmission in the Thalamocortical Motor Pathway

Rats were subchronically administrated either a ZNS-free or a therapeutic-relevant dose of ZNS (40 mg/kg/day) for 7 days. To explore the interaction between ZNS and GAP19 on glutamatergic transmission in the thalamocortical motor pathway (MoTN-M2C) of wild-type and S286L-TG, the perfusion medium in the M2C was commenced with MRS containing with or without 100 μM GAP19 (Cx43 inhibitor) (Figure 1B). The perfusate in the MoTN was commenced with MRS alone (Figure 1B). After the stabilization of L-glutamate levels in the M2C, the perfusate in the MoTN was switched from MRS to MRS containing 100 μM AMPA (AMPA/glutamate receptor agonist) for 180 min (Figure 1B).

#### 2.3.3. Study_3: Effects of Subchronic Administration of the Therapeutic-Relevant Dose of ZNS and Acute Local Administration of GAP19 into the M2C on Repetitive Potassium-Dependent L-Glutamate Release in the M2C

Study_3 was composed of two experimental designs, Study_3-1 and Study_3-2. Rats were subchronically administrated by either a ZNS-free or therapeutic-relevant dose of ZNS (40 mg/kg/day) for 7 days (Study_3-1). To explore the effects of subchronic administration of therapeutic-relevant doses of ZNS on Cx43 associated transmission in the M2C induced by repetitive potassium-evoked stimulations of wild-type and S286L-TG, the perfusion medium in the M2C was commenced with MRS. After the stabilization of L-glutamate levels in the M2C, the perfusate in the M2C was switched to HKMRS for 20 min (1^st^ potassium-evoked stimulation: HKMRS 1^st^) (Figure 1C). After the 1^st^ potassium-evoked stimulation, the perfusate in the M2C was returned to MRS for 240 min (recovery) (Figure 1C). After the recovery, perfusate in the M2C was switched to HKMRS (2^nd^ potassium-evoked stimulation: HKMRS 2^nd^) for 20 min (Figure 1C). After the 2^nd^ potassium-evoked stimulation, the perfusate in the M2C was returned to MRS again (Figure 1C).

To explore the effects of local administration of GAP19 (Cx43 inhibitor) ZNS on Cx43 associated transmission in the M2C induced by repetitive potassium-evoked stimulations of wild-type and S286L-TG (Study_3-2), the perfusion medium in the M2C was commenced MRS containing with or without 100 μM GAP19 (Figure 1C). After the stabilization of L-glutamate levels in the M2C, the perfusate in the M2C was switched to HKMRS containing the same agent for 20 min (1^st^ potassium-evoked stimulation) (Figure 1C). After the 1^st^ potassium-evoked stimulation, the perfusate in the M2C was returned to MRS containing the same agent for 240 min (Figure 1C). After the recovery, perfusate in the M2C was switched to HKMRS containing the same agent (2^nd^ potassium-evoked stimulation) for 20 min (Figure 1C). After the 2^nd^ potassium-evoked stimulation, the perfusate in the M2C was returned to MRS containing the same agent again (Figure 1C).

### 2.4. Ultra-High-Performance Liquid-Chromatography (UHPLC)

L-glutamate levels were determined using UHPLC equipped with xLC3185PU (Jasco, Tokyo, Japan) and fluorescence detection (xLC3120FP, Jasco, Tokyo, Japan) following dual derivatisation with isobutyryl-L-cysteine and o-phthalaldehyde [45,46]. Derivatised solutions were prepared by dissolving isobutyryl-L-cysteine (2 mg) and o-phthalaldehyde (2 mg) in 0.1 mL ethanol, followed by the addition of 0.9 mL sodium borate buffer (0.2 M, pH 9.0) [47]. Automated pre-column derivatisation was performed by drawing 5 μL aliquots of sample, standard, or blank solutions and 5 μL of derivatisation solution together into a reaction vial and incubating for 5 min before injection. The derivatised samples (5 μL aliquots) were injected by an auto sampler (xLC3059AS, Jasco). The analytical column (YMC Triat C18, particle 1.8 μm, 50 × 2.1 mm, YMC, Kyoto, Japan) was maintained at 45 °C and flow rate was set at 500 μL/min. A linear gradient elution program was performed over a period of 10 min with mobile phases A (0.05 M citrate buffer, pH 5.0) and B (0.05 M citrate buffer containing 30% acetonitrile and 30% methanol, pH 3.5). The excitation/emission wavelengths of the fluorescence detector were set at 280/455 nm.

### 2.5. Preparation of Primary Astrocyte Culture

Astrocytes were prepared using a protocol adapted from previously described methods [47,48,49]. Pregnant Sprague-Dawley rats (SLC, Sizuoka, Japan) were housed individually in cages, kept in air-conditioned rooms (temperature, 22 ± 2 °C) set at 12 h light/dark cycle, with free access to food and water. Cultured astrocytes were prepared from cortical astrocyte cultures of neonatal Sprague-Dawley rats (N = 48) sacrificed by decapitation at 0–24 h of age. The cerebral hemispheres were removed under dissecting microscope. Tissue was chopped into fine pieces using scissors and then triturated briefly with a micropipette. Suspension was filtered using 70 µm nylon mesh (BD, Franklin Lakes, NJ, USA) and centrifuged. Pellets were then re-suspended in 10 mL Dulbecco’s modified Eagle’s medium containing 10% fetal calf serum (fDMEM), which was repeated three times. After culture for 14 days (DIV14), contaminating cells were removed by shaking in a standard incubator (BNA-111, Espec, Osaka, Japan) for 16 h at 200 rpm. On DIV21, astrocytes were removed from flasks by trypsinization and seeded directly onto translucent PET membrane (1.0 μm) with 24-well plates (BD) at a density of 1 × 105 cells/cm^2^ for experiments. From DIV21 to DIV28, the culture medium (fDMEM) was changed twice a week, and nicotine (0, 1 and 3 μM) or ZNS (50 μM) were added for subchronic administrations (7 days). On DIV28, cultured astrocytes were washed out using ACSF, and this was repeated three times. The ACSF comprised NaCl 150.0 mM, KCl 3.0 mM, CaCl_2_ 1.4 mM, MgCl_2_ 0.8 mM, and glucose 5.5 mM, buffered to pH 7.3 with 20 mM HEPES buffer. The remaining adherent cells contained 95% GFAP-positive and A2B5-negative cells detected using immunohistochemical staining [47,48,49]. After the wash out, plasma membrane proteins of cultured astrocytes were extracted using Minute Plasma Membrane Protein Isolation Kit (Invent Biotechnologies, Plymouth, MN, USA).

### 2.6. Simple Western Analysis

Total plasma membrane proteins of rat frontal cortex and primary cultured astrocytes were extracted using Minute Plasma Membrane Protein Isolation Kit (Invent Biotechnologies, Plymouth, MN, USA) [33]. Simple Western analyses were performed using Wes (ProteinSimple, Santa Clara, CA, USA) according to the ProteinSimple user manual [21,33]. Lysate of primary cultured astrocytes were mixed with a master mix (ProteinSimple, San Jose, CA, USA) to a final concentration of 1 × sample buffer, 1 × fluorescent molecular weight marker and 40 mM dithiothreitol, then heated at 95 °C for 5 min. The samples, blocking reagent, primary antibodies, HRP-conjugated secondary antibodies, chemiluminescent substrate, separation and stacking matrices were also dispensed to designated wells in a 25-well plate. After plate loading, the separation electrophoresis and immunodetection steps took place in the capillary system and were fully automated. Simple Western analysis was carried out at room temperature, and instrument default settings were used. Capillaries were first filled with separation matrix followed by stacking matrix, and about 40 nL sample loading. During electrophoresis, proteins were separated on the basis of molecular weight through the stacking and separation matrices at 250 volts for 40–50 min and then immobilized on the capillary wall using proprietary photo-activated capture chemistry. The matrices were then washed out. Capillaries were next incubated with a blocking reagent for 15 min, and target proteins were immunoprobed with primary antibodies followed by HRP-conjugated secondary antibodies. Antibodies of GAPDH (NB300-322SS, Novus Biologicals, Littleton, CO, USA) and Cx43 (C6219, Sigma-Aldrich, St. Louis, MO) were diluted in antibody diluent (ProteinSimple) with 1:100 dilution. The antibody incubation time was 0–120 min with antibody diluents. Luminol and peroxide (ProteinSimple) were then added to generate chemiluminescence which was captured by a CCD camera. The digital image was analyzed with Compass software (ProteinSimple), and the quantified data of the detected protein were reported as molecular weight, signal/peak intensity.

### 2.7. Data Analysis

All experiments in this study were designed with equally sized animal groups (N = 6) without carrying out a formal power analysis, in keeping with previous studies [21,22,23,33,50,51,52]. All values are expressed as mean ± standard deviation (SD) and *P* < 0.05 (two-tailed) was considered statistically significant for all tests. Drug levels in acutely local and subchronically systemic administrations were selected based on values in previous studies [21,22,23,24,33,50,51,52]. Where possible, we sought to randomize and blind the data. In particular, for the determination of extracellular transmitter levels, the sample order on the autosampler was determined by a random number table.

Regional transmitter concentrations were analyzed by Mauchly’s sphericity test followed by (MANOVA) using BellCurve for Excel ver. 3.2 (Social Survey Research Information Co., Ltd., Tokyo, Japan). When the data did not violate the assumption of sphericity (*P* > 0.05), the F-value of MANOVA was analyzed using sphericity assumed degrees of freedom. However, if the assumption of sphericity was violated (*P* < 0.05), the F-value was analyzed using Chi-Muller’s corrected degrees of freedom. When the F-value for the genotype/drug/time factors of MANOVA was significant, the data was analyzed by Tukey’s post hoc test. Transmitter level was expressed as the area under the curve between 20 and 180 min (AUC20–180) after perfusion of target agent.

Protein expression of Cx43 in the M2C plasma membrane fraction was analysed by student T-test, one-way or two-way analysis of variance (ANOVA) with Tukey’s post hoc test using BellCurve for Excel. All statistical analyses complied with the recommendations on experimental design and analysis in pharmacology [53].

### 2.8. Nomenclature of Targets and Ligands

Key protein targets and ligands in this article are hyperlinked to corresponding entries in http://www.guidetopharmacology.org, which is the common portal for data from the IUPHAR/BPS Guide to PHARMACOLOGY [54] and are permanently archived in the Concise Guide to PHARMACOLOGY 2017/18 [55].

## 3. Results

### 3.1. Cx43 Expression in the M2C Plasma Membrane Fraction of S286L-TG and Its Response to Subchronic Nicotine Administration In Vivo

**Figure 2:**
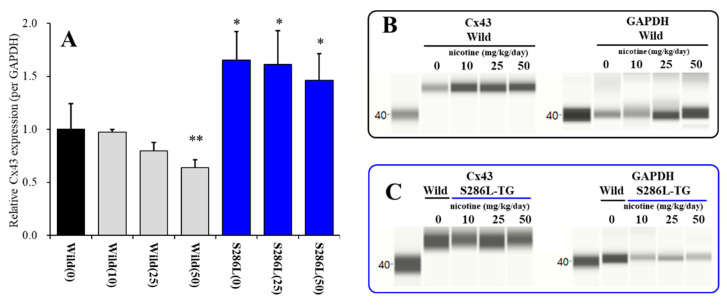
Dose-dependent effects of subcutaneous administration of subchronic doses of nicotine (0, 10, 25 and 50 mg/kg/day) for 7 days on Cx43 expression in the M2C plasma membrane fraction of wild-type and S286L-TG (**A**) and pseudo-gel images using Simple Western using anti-GAPDH and anti-Cx43 antibody for blotting of plasma membrane fractions of wild-type (**B**) and S286L-TG rats (**C**). In panel 2A, ordinate: mean ± SD (n = 6) of relative protein level of Cx43. *P < 0.05, **P < 0.01 relative to nicotine free wild-type by one-way analysis of variance (ANOVA) with Tukey’s post hoc test.

An *in vitro* study demonstrated that Cx43 expression in the human umbilical vein endothelial cells was reduced by the subchronic administration of nicotine [24]. The present study also demonstrated that systemic subchronic administration of nicotine (10, 25 and 50 mg/kg/day) [41] for 7 days, dose-dependently decreased Cx43 expression level in the M2C (ADSHE focus) plasma membrane fraction of wild-type (F_nicotine_(3,23) = 9.7 (*P* < 0.01)) (Figure 2A,B). Contrary to wild-type, neither subchronic nicotine administration of 25 nor 50 mg/kg/day could affect Cx43 expression level in the M2C plasma membrane fraction of S286L-TG, but Cx43 expression of S286L-TG was larger than compared with that of wild-type (F_nicotine_(3,23) = 8.2 (*P* < 0.01)) (Figure 2A,C). These results suggest that activation of α4β2-nAChR decreases Cx43 expression in plasma membrane, whereas S286L-mutant α4β2-nAChR impairs suppression effect on Cx43.

### 3.2. Effects of Subchronic Administration of Nicotine and ZNS on Cx43 Expression in Astroglial Plasma Membrane Fractions of Wild-Type Primary Cultured Astrocytes

**Figure 3:**
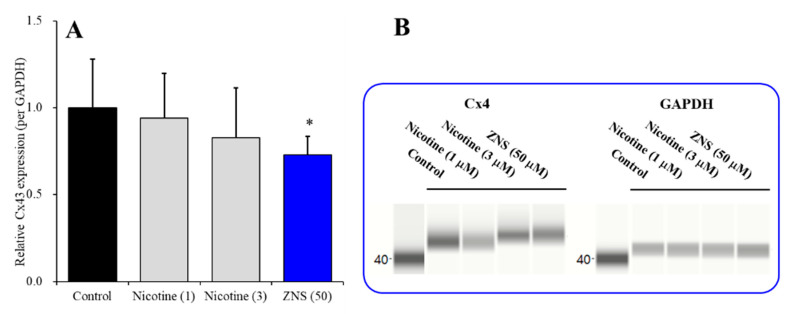
Concentration-dependent effects of subchronic administration of nicotine (0, 1 and 3 μM) and lowest therapeutic-relevant concentration of zonisamide (ZNS) (50 μM) for 7 days on Cx43 expression in the plasma membrane fraction of primary cultured astrocytes from wild-type rat (**A**) and pseudo-gel images using Simple Western results using anti-GAPDH and anti-Cx43 antibody for blotting of plasma membrane fractions of wild-type (**B**). In panel 3A, ordinate: mean ± SD (n = 6) of relative protein level of Cx43. *: *P* < 0.05 relative to control by student T-test. Concentration-dependent effects of nicotine on Cx43 expression was not detected by one-way ANOVA.

Cx43 is the most predominant connexin isoform in astrocytes but not expressed in neurons [28,29]; however, α4-nAChR expresses in neurons but not astrocytes [23]. Therefore, to clarify the target cell of the inhibitory effect of nicotine on Cx43 expression in plasma membrane, the effects of subchronic administration of nicotine (1 and 3 μM) for 7 days were examined in primary cultured astrocytes of wild-type rats. Cx43 expression in total lysate of the human umbilical vein endothelial cells was decreased to 50% by subchronic administration of 1 μM nicotine over 5 days [24]; however, in the present study, astroglial Cx43 in the plasma membrane fraction was not affected by subchronic nicotine (1 and 3 μM) administration (Figure 3A,B). Therefore, the inhibitory effects of nicotine on Cx43 expression in astroglial plasma membrane is not a direct action on astroglial Cx43 metabolism, but is possibly mediated by neuronal α4β2-nAChR indirectly.

Contrary to nicotine, ZNS directly decreases astroglial Cx43 expression in the astroglial plasma membrane, since the lowest therapeutic-relevant concentration of ZNS (50 μM) [38,40] decreased Cx43 expression in the plasma membrane fraction of primary cultured astrocytes (Figure 3A,B).

### 3.3. Effects of Subchronic Administration of Therapeutic-Relevant Dose of ZNS on Cx43 Expression in the M2C Plasma Membrane of Wild-Type and S286L-TG

**Figure 4:**
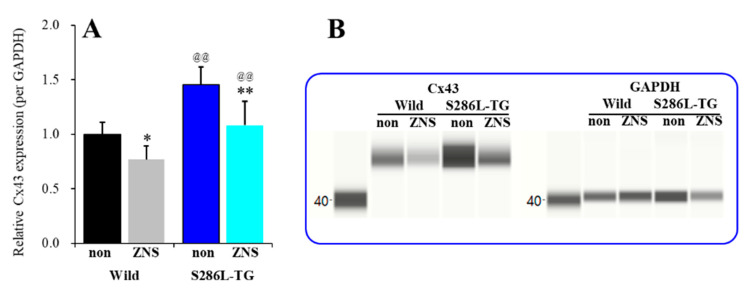
Effects of systemic subchronic administration of ZNS (40 mg/kg/day for 7 days) on Cx43 expression in the M2C plasma membrane fraction of wild-type and S286L-TG (**A**) and pseudo-gel images using Simple Western results using anti-GAPDH and anti-Cx43 antibody for blotting of plasma membrane fractions (**B**). In panel 4A, ordinate: mean ± SD (n = 6) of relative protein level of Cx43. *P < 0.05, **P < 0.01 vs. ZNS-free (non) and @@P < 0.01 vs. wild-type by two-way analysis of variance (ANOVA) with Tukey’s post hoc test.

Systemic subchronic administration of therapeutic-relevant doses of ZNS (40 mg/kg/day) for 7 days [23,39,40,44] decreased the Cx43 expression levels in the M2C plasma membrane fraction of both wild-type and S286L-TG (F_genotype_(1,20) = 35.2 (*P* < 0.01), F_ZNS_(1,20) = 21.6 (*P* < 0.01), F_genotype*ZNS_ (1,20) = 1.2 (*P* > 0.1)). The level of Cx43 in the M2C plasma membrane fraction of S286L-TG was larger than that of wild-type rats (Figure 4A). Subchronic administration of therapeutic-relevant dose of ZNS (40 mg/kg/day) for 7 days decreased Cx43 expression in the M2C plasma membrane fraction of both wild-type and S286L-TG (Figure 4A,B). Therefore, in S286L-TG, nicotine lost its suppression effect on Cx43 expression in astroglial plasma membrane due to the loss-of-function S286L-mutant α4β2-nAChR, but ZNS directly decreased Cx43 levels in the astroglial plasma membrane without mediating neuronal function.

### 3.4. Microdialysis Study

#### 3.4.1. Effects of Local Administration of RJR2403 into the RTN and Subchronic Administration of Therapeutic-Relevant Dose of ZNS on the L-Glutamate Release in the M2C Induced by Local Administration of AMPA into the MoTN (Study_1)

**Figure 5:**
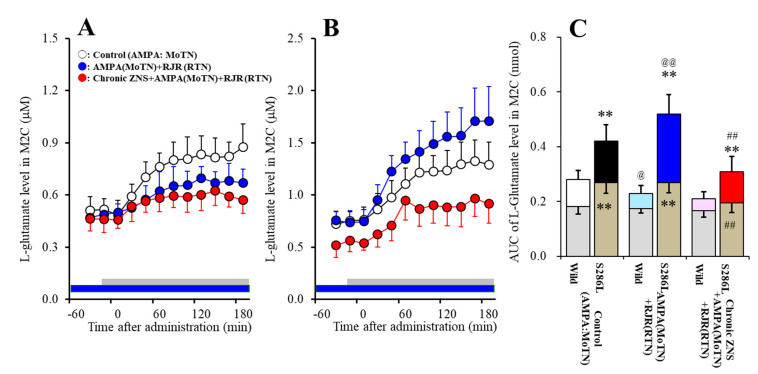
Effects of local administration of RJR2403 (α4β2-nAChR agonist) into the reticular thalamic nucleus (RTN) and subchronic administration of therapeutic-relevant dose of ZNS (40 mg/kg/day for 7 days) on the L-glutamate release in the secondary motor cortex (M2C) induced by local administration of 100 μM amino-3-(3-hydroxy-5-methyl-isoxazol-4-yl)propanoic acid (AMPA) into the motor thalamic nuclei (MoTN) of wild-type (**A**) and S286L-TG (**B**). Rats were subchronically administered a therapeutic-relevant dose of ZNS (chronic ZNS) or ZNS-free (control or AMPA+RJR) using a subcutaneous osmotic pump. In ZNS non-treated rats, perfusion medium in the RTN was commenced with modified Ringer’s solution (MRS) with (AMPA+RJR: blue circles) or without (control: opened circles) 100 μM RJR2403 (blue bars). After the stabilization of L-glutamate levels in the M2C, the perfusion medium in the MoTN was switched from MRS to MRS containing 100 μM AMPA for 180 min (gray bars). In ZNS treated rats (chronic ZNS: red circles), perfusion medium in the RTN was commenced with MRS containing 100 μM RJR2403. After the stabilization of L-glutamate levels in the M2C, the perfusion medium in the MoTN was switched from MRS to MRS containing 100 μM AMPA for 180 min (gray bars). Ordinates indicate mean extracellular L-glutamate level (μM) (N = 6), and abscissas indicate time after AMPA-evoked stimulation (min). Blue bars indicate the perfusion with 100 μM RJR2403 into the RTN. Gray bars indicate the perfusion with 100 μM AMPA into the MoTN. (**C**) indicates the area under curve value (AUC) value of extracellular L-glutamate level (nmol) before (basal extracellular L-glutamate level) and during perfusion with AMPA (from 20 to 180 min) of Figure 5A and 5B. Gray columns in Figure 5C indicate the AUC values of basal extracellular levels of L-glutamate in Figure 5A,B (during −60 to 0 min). **P < 0.01; relative to wild-type, @P < 0.05, @@P < 0.01; relative to control, ##P < 0.01; relative to AMPA+RJR by multivariate analysis of variance (MANOVA) with Tukey’s post hoc test.

Study_1 demonstrated varying responses between wild-type and S286L-TG to the local administration of 100 μM RJR2403 (α4β2-nAChR agonist) into the RTN on L-glutamate release in the M2C that was induced by MoTN AMPA-evoked stimulation (F_genotype_(1,20) = 102.2 (*P* < 0.01), F_agent_(1,20) = 1.1 (*P* > 0.1), _Fgenotype*agent_(1,20)=11.2 (*P* < 0.01), F_time_(3.1,62.1) = 110.9 (*P* < 0.01), F_genotype*agent*time_(3.1,62.1) = 6.7 (*P* < 0.01)) (Figure 5A–C). Perfusion with 100 μM AMPA into the MoTN increased extracellular L-glutamate levels in the M2C (AMPA-evoked L-glutamate release) of both wild-type and S286L-TG [21,22] (Figure 5A–C). In concurrence with previous studies, basal extracellular L-glutamate in the M2C of S286L-TG was larger than that of wild-type (Figure 5C) [21,22,23]. Neither basal extracellular L-glutamate levels in the M2C of wild-type nor S286L-TG were affected by perfusion with 100 μM RJR2403 into the RTN (Figure 5A–C). Contrary to basal levels, perfusion with 100 μM RJR2403 into the RTN decreased AMPA-evoked L-glutamate release in the M2C of wild-type rats; however, in S286L-TG, local administration of RJR2403 into the RTN increased AMPA-evoked L-glutamate release in the M2C of S286L-TG (Figure 5A–C).

Study_1 also revealed the statistically significant effects of systemic subchronic administration of therapeutic-relevant doses of ZNS (40 mg/kg/day for 7 days) on L-glutamate release in the M2C induced by MoTN AMPA-evoked stimulation (F_genotype_(1,20) = 58.6 (*P* < 0.01), F_agent_(1,20) = 30.7 (P < 0.01), F_genotype*agent_(1,20) = 21.1 (*P* < 0.01), F_time_(3.2,64.1) = 82.7 (*P* < 0.01), F_genotype*agent*time_(3.2,64.1 )= 6.7 (*P* < 0.01)) (Figure 5A–C). Subchronic administration of therapeutic-relevant doses of ZNS did not affect basal extracellular L-glutamate levels in the M2C of wild-type but decreased that of S286L-TG (Figure 5A–C). Subchronic administration of therapeutic-relevant doses of ZNS inhibited the AMPA-evoked L-glutamate release in the M2C of S286L-TG without affecting that of wild-type rats (Figure 5A–C).

#### 3.4.2. Interaction between Local Administration of GAP19 into the M2C and the Subchronic Administration of Therapeutic-Relevant Dose of ZNS on AMPA-Evoked L-Glutamate Release in the M2C (Study_2)

**Figure 6:**
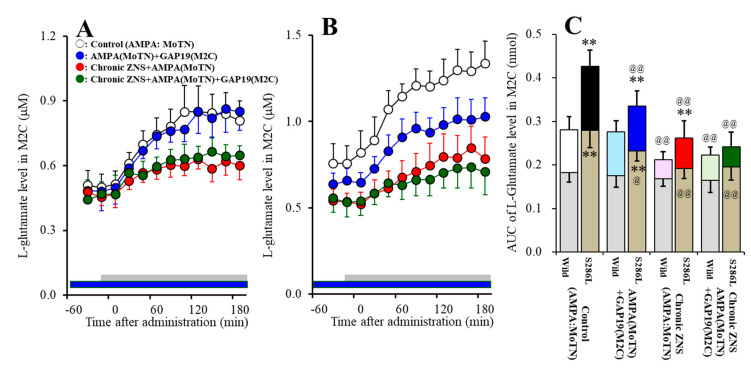
Interaction between local administration of GAP19 (Cx43 inhibitor) into the M2C and subchronic administration of therapeutic-relevant dose of ZNS (40 mg/kg/day for 7 days) on AMPA-evoked L-glutamate release in the M2C of wild-type (**A**) and S286L-TG (**B**). Rats were subchronically administered a therapeutic-relevant dose of ZNS (chronic ZNS or Chronic ZNS+GAP19) or ZNS free (control or AMPA + GAP19) using a subcutaneous osmotic pump. Perfusion medium in the M2C was commenced with MRS containing with (AMPA+GAP19 and chronic ZNS+GAP19) or without (control and chronic ZNS) 100 μM GAP19. After the stabilization of L-glutamate level in the M2C, the perfusion medium in the MoTN was switched from MRS to MRS containing 100 μM AMPA for 180 min. Ordinates indicate mean extracellular L-glutamate level (μM) (N = 6), and abscissas indicate time after AMPA-evoked stimulation (min). Blue bars indicate the perfusion with 100 μM GAP19 into the M2C. Gray bars indicate the perfusion with 100 μM AMPA into the MoTN. (**C**) indicates the AUC value of extracellular L-glutamate level (nmol) before (basal extracellular L-glutamate level) and during perfusion with AMPA (from 20 to 180 min) of Figure 6A,B. Gray columns in Figure 6C indicate the AUC values of basal extracellular levels of L-glutamate in Figure 6A,B. **P < 0.01; relative to wild-type, @*P* < 0.05, @@*P* < 0.01; relative to control by MANOVA with Tukey’s post hoc test.

Study_2 demonstrated the different responses between wild-type and S286L-TG to local administration of GAP19 (Cx43 inhibitor) into the M2C on L-glutamate release in the M2C induced by MoTN AMPA-evoked stimulation (F_genotype_(1,20) = 60.1 (*P* < 0.01), F_agent_(1,20) = 12.6 (*P* < 0.01), F_genotype*agent_(1,20) = 10.1 (*P* < 0.01), F_time_(6.5,130.0) = 351.4 (*P* < 0.01), F_genotype*agent*time_ (6.5,130.0) = 5.9 (*P* < 0.01)) (Figure 6A–C). Perfusion with 100 μM GAP19 into the M2C decreased both the basal extracellular L-glutamate level and AMPA-evoked L-glutamate release in the M2C of S286L-TG, whereas neither AMPA-evoked L-glutamate release nor basal extracellular L-glutamate levels in the M2C of wild-type were affected by perfusion with 100 μM GAP19 into the M2C (Figure 6A–C).

Systemic subchronic administration of therapeutic-relevant doses of ZNS (40 mg/kg/day for 7 days) decreased AMPA-evoked L-glutamate release without affecting basal extracellular L-glutamate level in the M2C of wild-type, whereas subchronic ZNS administration decreased both AMPA-evoked L-glutamate release and basal extracellular L-glutamate level in the M2C of S286L-TG (F_genotype_(1,20) = 52.2 (*P* < 0.01), F_agent_(1,20) = 72.3 (*P* < 0.01), F_genotype*agent_(1,20) = 13.3 (*P* < 0.01), F_time_(6.7,132.9) = 238.1 (*P* < 0.01), F_genotype*agent*time_ (6.7,132.9) = 3.7 (*P* < 0.01)) (Figure 6A–C).

In both ZNS treated wild-type and S286L-TG, perfusion with 100 μM GAP19 into the M2C did not affect either AMPA-evoked L-glutamate release or basal extracellular L-glutamate levels in the M2C (Figure 6A–C).

### 3.5. Effects of Subchronic Administration of Therapeutic-Relevant Dose of ZNS on Repetitive Potassium-Evoked L-Glutamate Release in the M2C (Study_3-1)

**Figure 7:**
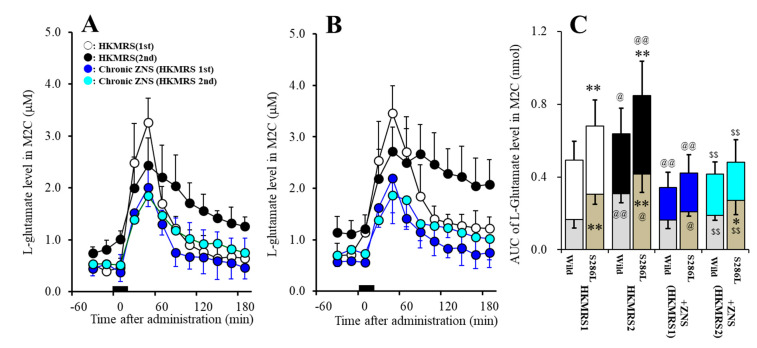
Effects of subchronic administration of therapeutic-relevant dose of ZNS (40 mg/kg/day for 7 days) on repetitive potassium-evoked L-glutamate release in the M2C of wild-type (**A**) and S286L-TG (**B**). Rats were subchronically administered with therapeutic-relevant dose of ZNS or ZNS free. Perfusion medium in the M2C was commenced with MRS. After the stabilization of L-glutamate level in the M2C, the perfusion medium was switched from MRS to HKMRS for 20 min (1^st^ potassium-evoked stimulation) (ZNS non-treated with 1^st^ stimulation: opened circles, ZNS administered with 1^st^ stimulation: red circles). After the 1^st^ potassium-evoked stimulation, the perfusate was returned to MRS for 240 min (recovery). Following recovery, the perfusate was switched to HKMRS for 20 min again (2^nd^ potassium-evoked stimulation). After the 2^nd^ potassium-evoked stimulation, perfusate was returned to MRS. Ordinates indicate mean extracellular L-glutamate level (μM) (N = 6), and abscissas indicate time after 1^st^ or 2^nd^ potassium-evoked stimulations (min). Black bars indicate the perfusion with HKMRS (1^st^ and 2^nd^ potassium-evoked stimulation). (**C**) indicates the AUC value of extracellular L-glutamate level (nmol) before (basal extracellular L-glutamate level) and after potassium-evoked stimulation (from 20 to 180 min) of Figure 7A,B. Gray columns in Figure 7C indicate the AUC values of basal extracellular levels of L-glutamate in Figure 7A,B. *P < 0.05, **P < 0.01; relative to wild-type, @*P* < 0.05, @@*P* < 0.01; relative to HKMRS (1^st^), $$*P* < 0.01 relative to HKMRS (2^nd^) by MANOVA with Tukey’s post hoc test.

Repetitive potassium-evoked stimulation in the M2C use-dependently increased basal extracellular L-glutamate level and potassium-evoked L-glutamate release in the M2C of wild-type and S286L-TG (F_genotype_(1,20) = 11.5 (*P* < 0.01), F_potassium_(1,20) = 7.4 (*P* < 0.05), F_genotype*potassium_(1,20) = 0.1 (*P* > 0.5), F_time_(3.4,67.4) = 162.0 (*P* < 0.01), F_genotype*potassium*time_ (3.4,67.4) = 2.1 (*P* > 0.1)) (Figure 7A–C). Basal extracellular L-glutamate level (before 1^st^ potassium-evoked stimulation) in the M2C of S286L-TG was higher than that of wild-type (Figure 7A–C). First potassium-evoked stimulation (perfusion with HKMRS into the M2C) increased extracellular L-glutamate level in the M2C (1^st^ potassium-evoked L-glutamate release) of both wild-type and S286L-TG, whereas potassium-evoked L-glutamate release was larger than that of wild-type (Figure 7A–C). After the 240 min recovery from the 1^st^ potassium-evoked stimulation, the basal extracellular L-glutamate levels were higher than basal L-glutamate level before the 1^st^ potassium-evoked stimulation of wild-type and S286L-TG (1^st^ vs. 2^nd^ in Figure 7A–C). Second potassium-evoked stimulation also increased L-glutamate release of both wild-type and S286L-TG (Figure 7A–C). Especially, the peak elevation of the 2^nd^ potassium-evoked L-glutamate release was lower than those of the 1^st^ potassium-evoked release, but the extracellular L-glutamate levels 180 min after the 2^nd^ potassium-evoked stimulation was larger than those after the 1^st^ potassium-evoked stimulation of both wild-type and S286L-TG (Figure 7A–C).

The subchronic administration of therapeutic-relevant doses of ZNS (40 mg/kg/day for 7 days) inhibited 1^st^ potassium-evoked L-glutamate release in the M2C of both wild-type and S286L-TG (F_genotype_(1,20) = 9.5 (*P* < 0.01), F_potassium_(1,20) = 20.4 (*P* < 0.01), F_genotype*potassium_(1,20) = 1.7 (*P* > 0.1), F_time_(3.8,76.8) = 230.5 (*P* < 0.01), F_genotype*potassium*time_(3.8,76.8) = 2.0 (*P* > 0.1)) (Figure 7A–C). Subchronic administration of therapeutic-relevant doses of ZNS decreased basal extracellular L-glutamate level (before 1^st^ potassium-evoked stimulation) in the M2C of S286L-TG without affecting that of wild-type (Figure 7A–C). Subchronic administration of therapeutic-relevant dose of ZNS decreased 1^st^ potassium-evoked L-glutamate release of both wild-type and S286L-TG (Figure 7A–C). After the subchronic administration of therapeutic-relevant dose of ZNS, the basal extracellular L-glutamate level and 1^st^ potassium-evoked L-glutamate release in the M2C between wild-type and S286L-TG became almost equal (Figure 7A–C).

The subchronic administration of therapeutic-relevant doses of ZNS inhibited 2^nd^ potassium-evoked L-glutamate release in the M2C of both wild-type and S286L-TG (F_genotype_(1,20) = 6.5 (*P* < 0.05), F_potassium_(1,20) = 28.0 (*P* < 0.01), F_genotype*potassium_(1,20) = 1.5 (*P* > 0.1), F_time_(5.2,104.6) = 78.6 (*P* < 0.01), F_genotype*potassium*time_(5.2,104.6) = 1.4 (*P* > 0.1)) (Figure 7A–C). Subchronic administration of therapeutic-relevant doses of ZNS decreased basal extracellular L-glutamate level after 1^st^ potassium-evoked stimulation in the M2C of both wild-type and S286L-TG, whereas the basal extracellular L-glutamate release after 1^st^ potassium-evoked stimulation of S286L-TG was larger than that of wild-type (Figure 7A–C). Subchronic administration of therapeutic-relevant doses of ZNS also decreased 2^nd^ potassium-evoked L-glutamate release of both wild-type and S286L-TG (Figure 7A–C).

Subchronic administration of therapeutic-relevant doses of ZNS inhibited repetitive potassium-evoked L-glutamate release in the M2C (F_genotype_(1,20) = 3.9 (*P* > 0.05), F_potassium_(1,20) = 3.1 (*P* > 0.05), F_genotype*potassium_(1,20) = 0.1 (*P* > 0.5), F_time_(4.0,80.3) = 111.8 (*P* < 0.01), F_genotype*potassium*time_(4.0,80.3) = 0.8 (*P* > 0.1)) (Figure 7A–C). There were no differences in basal extracellular L-glutamate levels or potassium-evoked L-glutamate releases in wild-type or S286L-TG between 1^st^ and 2^nd^ potassium-evoked stimulation (Figure 7A–C).

### 3.6. Effects of Local Administration of GAP19 on Repetitive Potassium-Evoked L-Glutamate Release in the M2C (Study_3-2)

**Figure 8:**
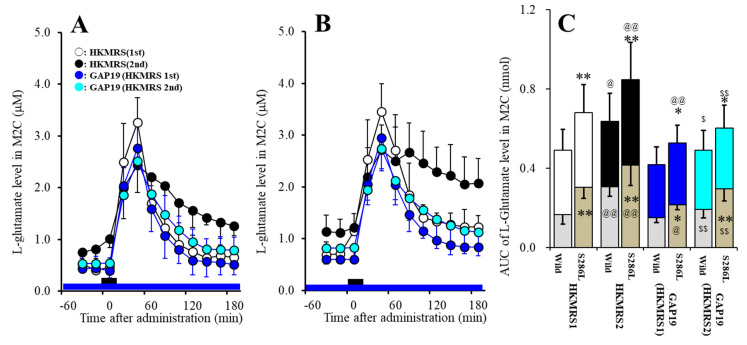
Effects of local administration of GAP19 (Cx43 inhibitor) into the M2C on repetitive potassium-evoked L-glutamate release in the M2C of wild-type (**A**) and S286L-TG (**B**). Perfusion medium in the M2C was commenced with MRS containing with or without 100 μM GAP19. After the stabilization of L-glutamate level in the M2C, the perfusion medium was switched from MRS to HKMRS containing the same agent for 20 min (1^st^ potassium-evoked stimulation) (GAP19 non-treated with 1^st^ stimulation: opened circles, GAP19 administered with 1^st^ stimulation: red circles). After the 1^st^ potassium-evoked stimulation, the perfusate was returned to MRS containing the same agent for 240 min (recovery). Following recovery, the perfusate was switched to HKMRS containing the same agent for 20 min again (2^nd^ potassium-evoked stimulation). After the 2^nd^ potassium-evoked stimulation, perfusate was returned to MRS containing the same agent (GAP19 non-treated with 2^nd^ stimulation: blue circles, GAP19 administered with 2^nd^ stimulation: green circles). Ordinates indicate mean extracellular L-glutamate level (μM) (N = 6), and abscissas indicate time after 1^st^ or 2^nd^ potassium-evoked stimulations (min). Black bars indicate the perfusion with HKMRS (1^st^ and 2^nd^ potassium-evoked stimulation). Blue bars indicate the perfusion with 100 μM GAP19 into the M2C. (**C**) indicates the AUC value of extracellular L-glutamate level (nmol) before (basal extracellular L-glutamate level) and after potassium-evoked stimulation (from 20 to 180 min) of Figure 8A,B. Gray columns in Figure 8C indicate the AUC values of basal extracellular levels of L-glutamate in Figure 8A,B. * *P* < 0.05, ** *P* < 0.01; relative to wild-type, @*P* < 0.05, @@ *P* < 0.01; relative to HKMRS (1^st^) and $ *P* < 0.05, $$ *P* < 0.01 relative to HKMRS (2^nd^) by MANOVA with Tukey’s post hoc test. Control data (HKMRS1 and HKMRS2) were same data of Study_3-1 (Figure 7).

Acute local administration of 100 μM GAP19 into the M2C inhibited 1^st^ potassium-evoked L-glutamate release in the M2C of S286L-TG without affecting that of wild-type (F_genotype_(1,20) = 11.5 (*P* < 0.01), F_potassium_(1,20) = 7.4 (*P* < 0.05), F_genotype*potassium_(1,20) = 0.1 (*P* > 0.1), F_time_(3.4,67.4) = 162.0 (*P* < 0.01), F_genotype*potassium*time_(3.4,67.4) = 2.1 (*P* > 0.1)) (Figure 8A–C). Local administration of 100 μM GAP19 into the M2C decreased basal extracellular L-glutamate level (before 1^st^ potassium-evoked stimulation) in the M2C of S286L-TG without affecting that of wild-type (Figure 8A–C). Local administration of GAP19 also decreased 1^st^ potassium-evoked L-glutamate release of S286L-TG without affecting that of wild-type (Figure 8A–C).

Local administration of GAP19 into the M2C inhibited 2^nd^ potassium-evoked L-glutamate release in the M2C of both wild-type and S286L-TG (F_genotype_(1,20) = 8.1 (*P* < 0.01), F_potassium_(1,20) = 3.7 (*P* > 0.05), F_genotype*potassium_(1,20) = 0.1 (*P* > 0.1), F_time_(2.6,51.1)=243.4 (*P* < 0.01), F_genotype*potassium*time_(2.6,51.1) = 0.3 (*P* > 0.1)) (Figure 8A–C). Local administration of GAP19 into the M2C decreased basal extracellular L-glutamate level after 1^st^ potassium-evoked stimulation in the M2C of both wild-type and S286L-TG, whereas the basal extracellular L-glutamate release after 1^st^ potassium-evoked stimulation of S286L-TG was larger than that of wild-type (Figure 8A–C). Local administration of GAP19 into the M2C also decreased 2^nd^ potassium-evoked L-glutamate release of both wild-type and S286L-TG (Figure 8A–C).

Local administration of GAP19 into the M2C inhibited repetitive potassium-evoked L-glutamate release in the M2C (F_genotype_(1,20) = 8.3 (*P* < 0.01), F_potassium_(1,20) = 12.3 (*P* < 0.01), F_genotype*potassium_(1,20) = 0.7 (*P* < 0.1), F_time_(4.5,88.9) = 117.3 (*P* < 0.01), F_genotype*potassium*time_(4.5,88.9) = 0.8 (*P* < 0.1)) (Figure 8A–C). There were no differences of basal extracellular L-glutamate levels or potassium-evoked L-glutamate releases in wild-type and S286L-TG between 1^st^ and 2^nd^ potassium-evoked stimulation (Figure 8A–C).

## 4. Discussion

### 4.1. Pathomechanism of ADSHE Seizures Associated with Cx43

Various gene mutations, in *CHRNA2, CHRNA4, CHRNB2, CHR, KCNT1* and *DEPDC5*, have been identified in the ADSHE pedigrees [2,5,6]; however, it has been established that ADSHE/SHE syndrome is uniform of motor seizures during mainly the non-rapid eye movement sleep phase [2,4,5,6]. In spite of the uniformity of ADSHE, there are inter-individual variations of seizure frequency and prognosis in the same ADSHE pedigree [2,4,5,6]. Furthermore, in a majority of ADSHE patients, the occurrence of an ADSHE seizure is generally followed by several seizures during the same night [4]. These inter- and intra-individual variations of clinical features of ADSHE suggest the possibility that the combination of genetic abnormality with event-related transmission abnormalities of genetically normal transmission regulating molecules (induced by genetic based pathological events) plays important roles in the development of pathomechanisms of ADSHE seizures (epileptogenesis and/or ictogenesis).

**Figure 9:**
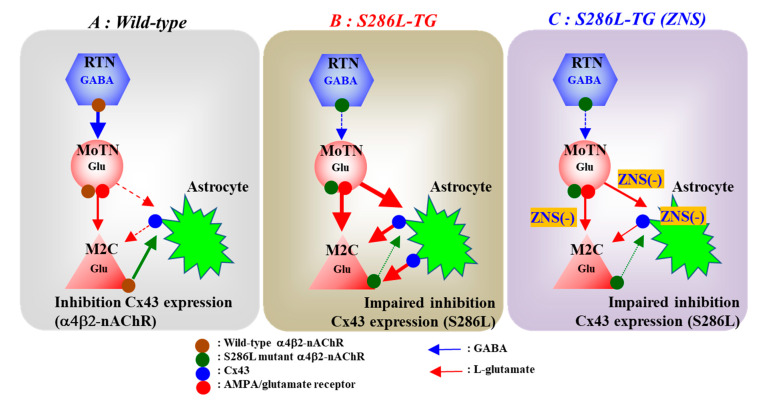
Proposed hypothesis of pathomechanisms and pathophysiology of S286L-TG. Proposed hypothesis for functional abnormalities of glutamatergic transmission in the thalamocortical pathways in wild-type (panel **A**), S286L-TG (panel **B**) and pathophysiology of ZNS-sensitive ADSHE seizure (panel **C**). RTN mainly projects GABAergic terminals to MoTN. Activation of α4β2-nAChR in the RTN enhances GABAergic inhibition in the RTN-MoTN pathways of wild-type (panel A). MoTN project glutamatergic terminals to the M2C. In the MoTN, both α4β2-nAChR and AMPA/glutamate receptor activate glutamatergic transmission to the M2C (panel **A**). Wild-type α4β2-nAChR suppresses the Cx43 expression in plasma membrane (panel A). Contrary to wild-type, loss-of-function S286L-mutant α4β2-nAChR attenuates the inhibition of Cx43 expression in the plasma membrane (panel B). Additionally, loss-of-function of S286L-mutant α4β2-nAChRs in RTN generates GABAergic disinhibition in the MoTN, leading to enhancement of glutamatergic transmission in M2C (panel **B**). Enhanced glutamatergic transmission in the M2C stimulates both glutamatergic pyramidal neurons and astrocytes resulting in activation of Cx43 (panel **B**). A combination of enhanced glutamatergic transmission and Cx43 expression in the M2C are candidate mechanisms of focus generation in the M2C (panel **B**). ZNS reduced Cx43 expression in the M2C (panel **C**). ZNS also compensates the enhanced glutamatergic transmission in the thalamocortical glutamatergic transmission via activation of II-mGluR [22] in the MoTN and GABA release [39,62] in the M2C [21].

We have already reported the several candidate pathomechanisms of ADSHE seizures associated with S284L-mutation using S286L-TG [21,22]. Impaired GABAergic inhibition in the intra-thalamic GABAergic pathway (RTN-MoTN) via enhanced desensitization of S286L-mutant α4β2-nAChR contributes to the pathomechanisms of all three major typical ADSHE seizures, ‘nocturnal paroxysmal dystonia’, ‘nocturnal paroxysmal arousal’ and ‘episodic nocturnal wandering’ (Figure 9B) [21,22]. The hyperactivation of the glutamatergic transmission in thalamocortical motor pathway (MoTN-M2C) induced by GABAergic disinhibition in the MoTN via impaired S286L-mutant α4β2-nAChR are integrated in the M2C resulting in the generation of ADSHE focus of episodic nocturnal wandering and nocturnal paroxysmal arousal [21] (Figure 9B). The hyperactivation of glutamatergic transmission in the thalamic hyperdirect pathway (MoTN-STN), also induced by the S286L-mutant α4β2-nAChR-associated GABAergic disinhibition, contributes to the pathomechanism of the other phenotype, nocturnal paroxysmal dystonia [22]. Therefore, the loss-of-functional abnormality of S286L-mutant α4β2-nAChR induced by a combination of enhanced sensitivity and desensitization to ACh [56,57] in the RTN plays fundamental roles in the ictogenesis of ADSHE patients with the S284L-mutation [21,22]. In spite of these efforts, the mechanisms of focus generation and increased seizure frequency following initial seizure in the same night remains to be clarified.

In our previous study, 50 mM potassium-evoked stimulation in the M2C increased L-glutamate release in both wild-type and S286L-TG, whereas the 50 mM potassium-evoked L-glutamate release of S286L-TG was almost equal to that of wild-type [21]. However, in the present study, 100 mM potassium-evoked L-glutamate release was larger compared with wild-type. These different responses of S286L-TG between 50 mM and 100 mM potassium-evoked stimulations suggest that S286L-TG does not acquire electrophysiological hypersensitivity in focus regions (M2C) [21], but acquires abnormality in extracellular potassium-sensitive L-glutamate release regulation systems. The neurotransmitter exocytosis is activated by 25 mM potassium-evoked stimulation [58], whereas the generation of astroglial gliotransmitter release requires higher than 50 mM potassium-evoked stimulation [47,49,59]. Additionally, astroglial L-glutamate release through astroglial connexin hemichannel is also activated by 100 mM potassium-evoked stimulation [33,34]. These demonstrations suggest the functional abnormalities of astroglial gliotransmitter release and/or astroglial hemichannel abnormalities of S286L-TG.

Abnormalities of several connexin isoforms associated with epilepsy have been reported [26]. Indeed, Cx43 expression is upregulated in the human epileptic tissue [60,61]. Recently, we have also demonstrated that the combination between increased Cx43 expression in the plasma membrane and extracellular potassium levels contributes to antipsychotic-induced convulsion [33]. Furthermore, subchronic administration of nicotine decreases Cx43 protein in the plasma membrane without affecting Cx43 mRNA in the human endothelial cells induced by the activation of posttranscriptional proteasomal protein degradation via α4β2-nAChR [24]. Based on clinical and preclinical evidence, to clarify the pathogenesis of ADSHE seizure associated with S284L-mutation, the present study determined Cx43-associated transmission in the thalamocortical motor pathway and focus regions in S286L-TG.

Confirming our hypothesis, the Cx43 expression in the M2C plasma membrane fraction of S286L-TG was upregulated compared with that of wild-type (Figure 9B). Interestingly, subchronic administration of nicotine dose-dependently reduced the Cx43 expression in the M2C plasma membrane of wild-type, but did not affect that of S286L-TG (Figure 9A,B). The upregulated Cx43 in focus region of S286L-TG provides at least two functional abnormalities of S286L-TG; increased basal L-glutamate level during resting stage, and enhanced glutamatergic transmission in thalamocortical motor pathway. Indeed, inhibition of Cx43 in the M2C by selective Cx43 inhibitor, GAP19 decreased basal extracellular L-glutamate level in the M2C of S286L-TG without affecting that of wild-type. Furthermore, local administration of GAP19 into the M2C also inhibited L-glutamate release induced by AMPA-evoke stimulation in the MoTN of S286L-TG without affecting that of wild-type. These results indicate that upregulated Cx43 in focus regions of S286L-TG is possibly induced by impaired inhibitory regulation of α4β2-nAChR (loss-of-functional S286L-mutant α4β2-nAChR). Therefore, the hypersensitive Cx43 hemichannel in the M2C plays important roles in the focus generation mechanism of S286L-TG (Figure 9B).

The present study reveals possible Cx43-associated mechanisms involved in the frequency of ADSHE seizures following the initial seizure during a single night. Repetitive potassium-evoked stimulation, of which intervals between 1^st^ and 2^nd^ potassium-evoked stimulation was set at 4 h, which is longer than the half-life of Cx43 (about 2–3 h) [63], use-dependently enhanced Cx43 hemichannel activity and thereby resulted in increased basal and depolarization-induced L-glutamate releases. These results suggest that ADSHE seizure activates glutamatergic transmission associated with the Cx43 hemichannel and maintains the epileptogenic condition over several hours. This hypothesis can also explain the frequent occurrence of ADSHE seizures during the non-rapid eye movement phase of sleep, since the sleep spindle, which is formed in the RTN and produces benign bursts, propagate through various cortical regions via intra-thalamic and thalamocortical pathways [64]. Therefore, sleep spindle burst during the non-rapid eye movement phase of sleep can be triggered in ADSHE seizures via the activation of upregulated Cx43 hemichannel activity.

The present study cannot explain the mechanisms by which neuron-specific α4β2-nAChR affects astrocyte specific Cx43 expression. Translated Cx43 folds in the endoplasmic reticulum and traffics to the plasma membrane as hemichannels or Gap-junctions following oligomerization in the Golgi. Ubiquitylation of Cx43 at the plasma membrane signals the internalization of Cx43 [28]. Internalization of Cx43 leads to annular Gap-junction or hemichannel endocytosis resulting in lysosome or autophagy degradation. Deubiquitylation of Cx43 rescues the protein from lysosomal degradation, allowing its recycling shift to the plasma membrane [28]. In future studies we will investigate the mechanisms of inhibitory regulation of α4β2-nAChR on metabolism and cellular localisation of astroglial Cx43 using immunohistochemistry. It is well known that single neuronal connexon docks associate with a connexon on an adjacent cell to form a Gap-junction channels [25,28]. Gap-junctions allow the passive diffusion of various intracellular molecules, including mRNA, transmitters, second messengers, ions and other regulatory substances involved in cell growth and differentiation [25,28]. Upregulated astroglial Cx43 S286L-TG should generate a GAP-junction with adjacent neuronal connexons resulting in the enhancement of communication between neurons and astrocytes via upregulated Gap-junctions. Exploring the detailed regulatory mechanisms of the suppressive function of neuron-specific α4β2-nAChR on the metabolism of astroglial specific Cx43 through neuro-astroglial communication is an important issue for understanding epileptogenesis. Furthermore, in this study, we used anti-GAPDH antibody for normalization of Cx43 expression level in the plasma membrane fraction. To study the detailed mechanisms of the suppressive effects of α4β2-nAChR on Cx43 metabolism in the plasma membrane, we shall use anti Na^+^/K^+^-ATPase antibody as a housekeeping protein of plasma membrane [65].

### 4.2. Pathophysiology of ADSHE Seizures Associated with ZNS

The present study demonstrated that subchronic administration of therapeutic-relevant dose of ZNS suppressed the enhanced glutamatergic transmission associated with Cx43 hemichannel of S286L-TG via reduction of Cx43 expression in the M2C plasma membrane. The present results suggest the loss-of-functional S286L-mutant nAChR impairs the inhibition of Cx43 degradation system, since subchronic administration of nicotine to S286L-TG could not decrease Cx43 expression (Figure 9B). A previous study reported that ZNS increased basal ACh release [44]; however, the enhancement of ACh release induced by ZNS cannot provide compensation of loss-of-functional S286L-mutant α4β2-nAChR due to two possibilities. The first, the enhanced ACh release probably cannot activate S286L-mutant α4β2-nAChR, since this mutation has enhanced sensitivity and desensitization to ACh [56,57]. Indeed, in the present study, application of nicotine did not affect Cx43 expression of S286L-TG. The second, CBZ, which cannot prevent ADSHE seizure of patients with S284L-mutation, increases basal ACh release, similar to ZNS [44,66]. Therefore, ZNS probably compensates the Cx43 metabolism dysfunction through an α4β2-nAChR independent system. Furthermore, the present study demonstrated that ZNS directly reduced Cx43 expression, since ZNS decreased Cx43 expression in plasma membrane fractions of primary cultured astrocytes. It has been well established that posttranscriptional processes, including phosphorylation, acetylation, nitrosylation, sumoylation and ubiquitylation, play important roles in the transport to the plasma membrane, intracellular communication and degradation of Cx43 [28]. ZNS suppressed endoplasmic reticulum stress induced cell death via increased human ubiquitin ligase 1 [67]. Taken together with these previous findings, subchronic administration of therapeutic-relevant dose and concentration of ZNS compensate the lack of inhibitory regulation systems associated by S286L mutant α4β2-nAChR of S286L-TG due to the activation of ubiquitin ligase (Figure 9C).

Although the present study did not determine the effects of ZNS on Cx43 hemichannel activity, established pharmacological profiles of ZNS suggest that ZNS possibly inhibits Cx43 hemichannel activity. Hemichannel activity is regulated by membrane potential, potassium, calcium ion concentration and intracellular pH. Depolarization, increased extracellular potassium ion levels, decreased extracellular calcium ion levels open hemichannel and enhanced gliotransmitter releases through hemichannel [33,34,68], whereas complete or partial closure of hemichannel can be triggered by low intracellular pH [69]. Both voltage-sensitive sodium channel and carbonic anhydrase inhibition is known to be one of the major mechanisms of anticonvulsive action of ZNS [62]. Inhibition of carbonic anhydrase reduces intracellular pH, and ZNS-induced acidosis and urolithiasis have been clinically reported [70]. Therefore, taken together, these pharmacological profiles of ZNS acutely and chronically inhibit L-glutamate release associated with upregulated and hyperactivated Cx43 hemichannel.

Subchronic administration of therapeutic-relevant dose of ZNS reduced Cx43 expression in the M2C plasma membrane and possibly inhibits Cx43 hemichannel activity (Figure 9C). The combination of these inhibitory effects of ZNS on Cx43 associated functions contributes to the compensation of functional abnormality of glutamatergic transmission of S286L-TG. The L-glutamate level in the M2C during resting stage and hyperactivated glutamatergic transmission in thalamocortical motor pathway were inhibited by ZNS (Figure 9C). These two inhibitions probably prevent the focus generation via a combination of reduced basal L-glutamate level with L-glutamate release induced by the hyperactivation of the focus generator and thalamocortical motor pathway activity. Furthermore, prolonged hyperactivation of Cx43 hemichannel activity induced by hyperactivation around focus regions were also prevented by the inhibition of various transmission regulation systems, including voltage-sensitive calcium ion channel, voltage-dependent sodium ion channel, calcium-induced calcium releasing system [37,38,39,44,58,71], induced by ZNS. This inhibition of secondarily activated Cx43 hemichannel activity by ZNS can explain the pathomechanisms of ADSHE seizures during the non-rapid eye movement phase of sleep and the reoccurrence of seizures during a single night [4].

The various antiepileptic/anticonvulsive mechanisms of ZNS have been established, including exocytosis monoamine, L-glutamate and GABA via direct/indirect modulation of voltage-dependent sodium channel, voltage-sensitive calcium channel, calcium-induced calcium releasing system, carbonic anhydrase, redox system, GABA receptor and metabotropic glutamate receptors [37,38,40,44,48,58,62,70,71,72]. In these various targets of ZNS, voltage-dependent sodium channel inhibition has been considered to be the major mechanism of antiepileptic/anticonvulsive actions of ZNS, similar to CBZ. The pathophysiology of CBZ-resistant/ZNS-sensitive ADSHE seizure associated with S284L-mutation indicates the interesting possibility that voltage-dependent sodium channel inhibition cannot contribute to antiepileptic/anticonvulsive action of ZNS against ADSHE seizures of S284L-mutation. Taken together with our previous findings, the present study provides several indirect compensations of loss-of-functional S284L-mutant α4β2-nAChR by ZNS via the activation of group II metabotropic glutamate receptors in M2C, MoTN and subthalamic nucleus [21,22,48], and reduced Cx43 expression in plasma membrane (Figure 9C). These two mechanisms possibly identify sodium channel inhibition-independent antiepileptic drugs as a potential therapeutic category. Furthermore, the present study suggests that ZNS can suppress Cx43 hemichannel activity. The detailed mechanisms of inhibitory effects of ZNS on Cx43 hemichannel should be determined in further studies, whereas inhibitory effects of ZNS on carbonic anhydrase and calcium-induced calcium releasing system are speculated to be candidate targets [37,62].

## 5. Conclusions

This study provided the primary pathomechanism and secondary pathophysiology of ADSHE seizures of ADSHE patients with S284L-mutation using S286L-TG as a valid ADSHE rat model. Loss-of-function of S286L-mutant α4β2-nAChR in the RTN produces paradoxical functional abnormalities of glutamatergic transmission in the thalamocortical motor pathway (RTN-MoTN-M2C). In the thalamocortical motor pathway of S286L-TG, intra-thalamic (RTN-MoTN) GABAergic inhibition is reversed to GABAergic disinhibition by S286L-mutant α4β2-nAChR in the RTN. The functional conversion of intra-thalamic inhibitory system leads to hyperactivation of the thalamocortical motor pathway. Additionally, Cx43 expression in the M2C plasma membrane of S286L-TG is upregulated and induced by a lack of inhibitory regulation of loss-of-functional S286L-mutant α4β2-nAChR. The upregulated Cx43 hemichannel probably contributes to the generation of ADSHE focus and unique ADSHE phenotypes of multiple seizures during the same night. Therapeutic-relevant doses of ZNS reduced Cx43 expression in the astroglial plasma membrane of both wild-type and S286L-TG. These compensations of ZNS on upregulated Cx43 expression in the ADSHE focus region play important roles in the CBZ-resistant/ZNS-sensitive ADSHE seizures of patients with S284L-mutation.
